# Association of Nutrition and Gut Microbiome Composition in Puerto Ricans with Alzheimer’s Disease Compared to Healthy Controls

**DOI:** 10.1002/alz.095665

**Published:** 2025-01-09

**Authors:** Cecilia Michelle Soler‐Llompart, Filipa Godoy‐Vitorino, Michel Santiago‐Berríos, Aidaliz Llorens‐ Bonilla, Hiram Morales, Carlos Alberto Herrero‐Rivera, Verónica Colon‐Pardo, Gerianne Olivieri‐Henry, Ana Cecilia Sala‐Morales, Vanessa Sepulveda

**Affiliations:** ^1^ University of Puerto Rico, Medical Sciences Campus, School of Medicine, San Juan, PR USA

## Abstract

**Background:**

Alzheimer’s disease (AD) is the most common cause of dementia, consisting of a highly debilitating disorder. It disproportionately affects Caribbean Hispanics, including Puerto Ricans living on the island, where it ranks as the fourth leading cause of death. Reported disparities in AD severity and mortality within this population may stem from various factors, including nutrition, lifestyle, socioeconomic risk status, and more prevalent chronic diseases. Evidence suggests that the gut microbiome plays a role in the pathophysiology of AD through neuroinflammation and amyloid deposition, leading to cognitive impairment. Diet also plays a role in modulating gut microbiome composition and diversity. Our study aims to examine how nutritional choices influence the microbiota composition and diversity of Puerto Ricans with AD compared to cognitively intact controls.

**Method:**

Forty participants (n = 40), 22 with AD and 18 controls, were clinically and cognitively assessed using Montreal Cognitive Assessment (MoCA). Stool samples were collected for genomic DNA extractions and microbiome characterization. A nutritional questionnaire was administered to participants.

**Result:**

While no significant differences were observed in the frequency of consumption of fruits and vegetables between AD vs. controls (p‐value 0.58), AD participants had a higher likelihood of consuming grains/ fiber more frequently (p‐value 0.05). There are no significant differences in bacterial diversity and richness between AD vs. controls regarding weekly legume, fruits, vegetables, and sweets intake. However, differences at the phylum taxa levels were observed. Cognitively intact participants with lower weekly consumption of sweets and increased consumption of fruits, vegetables, and legumes exhibited a higher abundance of Bacteroides.

**Conclusion:**

Diet choices with more frequent consumption of vegetables, fruits, and legumes and fewer sweets were associated with a higher abundance of Bacteroides in control participants. These bacteria are known for the production of butyrate, a compound with neuroprotective and anti‐inflammatory properties known to maintain homeostasis of the GI mucosa barriers. A better understanding of how nutritional choices influence the gut microbiome will be an invaluable approach to the development of modulation‐based therapeutic interventions to serve as part of a multidisciplinary approach to aim for better gut health in the AD population.

